# Replacement of long-segment ureteral defect with tapered demucosalized ileum: medium-term outcomes of 4 patients

**DOI:** 10.1186/s12894-023-01173-1

**Published:** 2023-01-07

**Authors:** Shulian Chen, Yu Jian, Wen Tang, Hao Gu, Kebing Luo, Denghao Yang, Huihui Xie, Guobiao Liang, Zeju Zhao

**Affiliations:** 1 Department of Urology, Affiliated Hospital of Zunyi Medical University, 149 Dalian Road, Zunyi, 563000 Guizhou China; 2 Department of Urology, People’s Hospital of Suiyang County, Guizhou Zunyi, China; 3 Department of Urology, The First People’s Hospital of Zunyi, Zunyi, Guizhou China

**Keywords:** Ureteral defects, Replacement, Tapered demucosalized ileum, Medium-term outcomes

## Abstract

**Purpose:**

Repair of long-segment ureteral defect (LSUD) is challenging. Currently available procedures carry some potential complications. We modified the ileal graft by tapering the wall and stripping the mucosa to combat associated pitfalls and first reported the medium-term outcomes of 4 patients.

**Material and methods:**

From September 2019 to October 2020, tapered demucosalized ileum (TDI) was used for LSUD reconstruction in 4 patients on the right (2 males and 2 females). Two patients were with panureteral avulsion and 2 with high-risk urothelial carcinoma in the distal ureter. TDI was made by tapering 1/2–2/3 of the antimesenteric ileal wall and stripping the mucosa with a blunt/blunt operating scissor. Follow-up modalities included serum creatinine, electrolytes, ultrasonography, CT urogram, renal scintigraphy, and ureteroscopy.

**Results:**

Mean operation time was 443 min (range 360–550) and blood loss was negligible. The mean follow-up period was 29 months (range 23–36). Vesicoureteral reflux and related pyelonephritis occurred in 1 patient, necessitating a repair operation (Clavien-Dindo grade IIIb). No strictures, obstructions, metabolic disorders, or electrolyte imbalances were observed in the remaining patients. In carcinoma patients, ureteroscopy in month 18 post-operation revealed ileal mucosal regrowth in the form of dwarf isolated islands. All renal units maintained adequate drainage and function during the follow-up.

**Conclusions:**

Ileal wall tapering and mucosa stripping confined to the muscularis mucosae level will not result in shrinkage, fibrosis, or stricture formation of the ileal ureter. The present work provides evidence for further application of TDI in the replacement of LSUD in patients.

## Background

Long-segment ureteral defect (LSUD) is a rare urological scenario, mainly caused by iatrogenic injuries, malignancy, tuberculosis, or extensive retroperitoneal fibrosis [1]. Reconstruction to restore ureteral continuity is challenging and probably involves substitution. Currently, available substitutes comprise the ileum, reconfigured colon, and appendix [2, 3]. Ileal ureter replacement has been widely used by urologists as its versatile usage, regardless of the defect length and side [4].

However, there are some intrinsic drawbacks of ileal substitutes, such as large tract lumen, mucous secretion, and metabolite absorption, which usually lead to pertinent complications like uroschesis, metabolic acidosis, electrolyte imbalance, and mucus obstruction [5]. Several modified techniques are proposed, including tapering the bowel graft, intestinal Onlay flap, and the Yong-Monti procedure, but only part of the issues is solved [6, 7].

To overcome the pitfalls completely, we modified the ileal conduit by tapering the antimesenteric part and stripping its mucosa to decrease ileal cross-sectional diameter and mucous secretion. In the short-term results of our preclinical animal research, which included 10 ureters of beagle dogs, no electrolyte disorders, severe obstruction, stricture, urine extravasation, or renal failure were observed, suggesting the feasibility and safety of the method. Intriguingly, H&E staining indicated no mucosal regeneration in 12 weeks [8]. Our current work aims to report this technique’s medium-term outcomes in 4 patients, including the patency of ileal ureter, renal function, electrolyte balance, and mucosa regeneration.

## Materials and methods

**Fig. 1:**
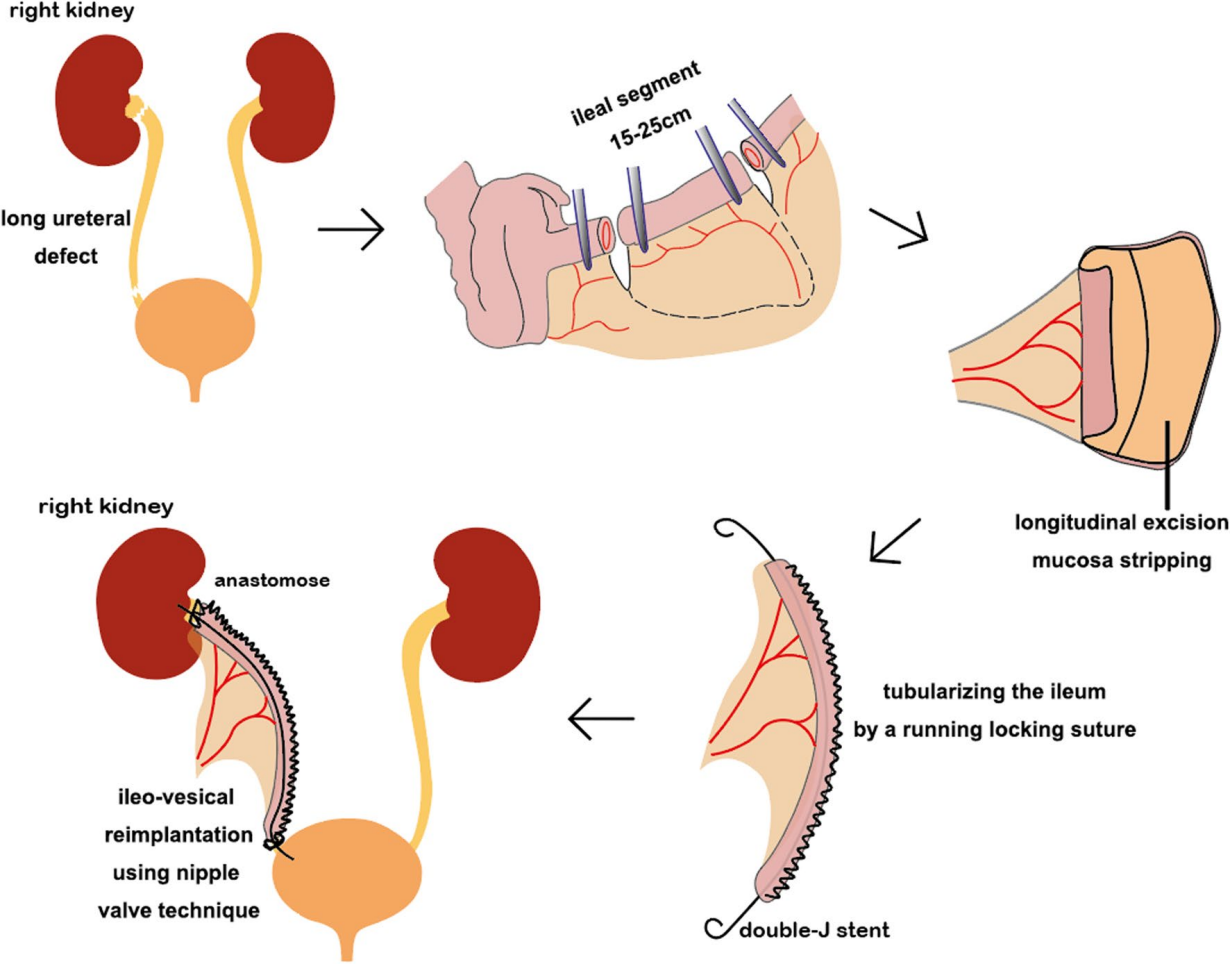
Schematic diagram of the surgery

From September 2019 to October 2020, TDI was used for long-segment ureteral reconstruction in 4 patients on the right (2 male and 2 female). Two patients presented with panureteral avulsion (1 due to uterine evacuation; 1 ureteroscopy). Another 2 cases presented with high-risk UTUC in the distal ureter (1 patient with T1G1, tumor size > 2 cm, and 1 with T2G2; Table 1). Ureter replacement was performed using TDI in all 4 cases. An ileal segment was isolated and 1/2 to 2/3 of the antimesenteric part was longitudinally resected. Ileal mucosa was stripped with the blade of a blunt/blunt operating scissor. Obvious bleeding was stopped by cauterization. A 12Fr red rubber catheter was placed in the detubularized ileum to support, thus expediting ensuing closure with a running locking suture of 3–0 vicryl. Then, a double J ureteral stent (7Fr) was inserted into the reconstructed ileal tract. The proximal anastomosis was performed on the renal pelvis or mid ureter with a simple interrupted suture of 3–0 vicryl. The ileal nipple valve technique was used in ileo-vesical reimplantation (Fig. 1). Two drainage tubes were placed around the anastomoses through separate incisions. Follow-up modalities included serum creatinine, electrolytes, ultrasonography, CT urogram, renal scintigraphy, and ureteroscopy in carcinoma patients. This study was approved by the Ethics Committee of Affiliated Hospital of Zunyi Medical University (Protocol No. 20190113) and consent was obtained from all the patients.

**Table 1:** Ureteral reconstruction: location and outcomes

Pt no	Age	Gender	Etiology	Length (cm)	Side&site	Follow-up (mos)	Creatine (mg/dl)	%Function
1	33	F	Uterine evacuation	28	Rt/panureter	30	0.76 → 0.83	42.11 → 29.55
2	45	F	Ureteral avulsion	23	Rt/panureter	27	0.85 → 0.71	39.37 → 40.87
3	75	M	Ureteral carcinoma	19	Rt/distal	36	2.65 → 2.02	53.01 → 58.24
4	42	M	Ureteral carcinoma	17	Rt/distal	23	0.84 → 1.04	58.74 → 56.57

## Results

**Fig. 2:**
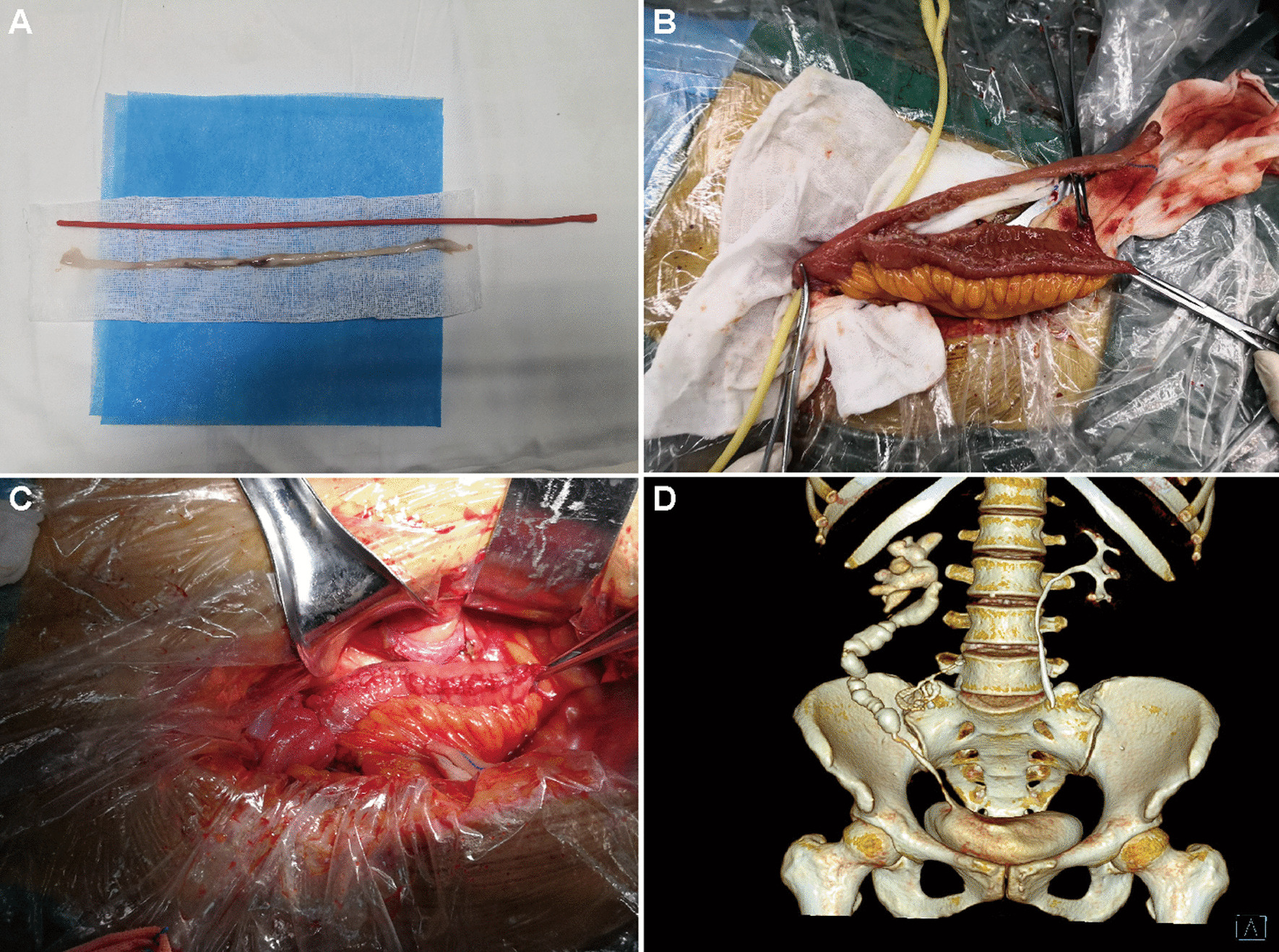
Images of patient 1. **A** Evacuated panureter approximately 28 cm in length. **B** Tapering of the isolated ileum. **C** Bridging the ureter by retubularized TDI. **D** CT urogram in month 30 post-surgery

**Fig. 3:**
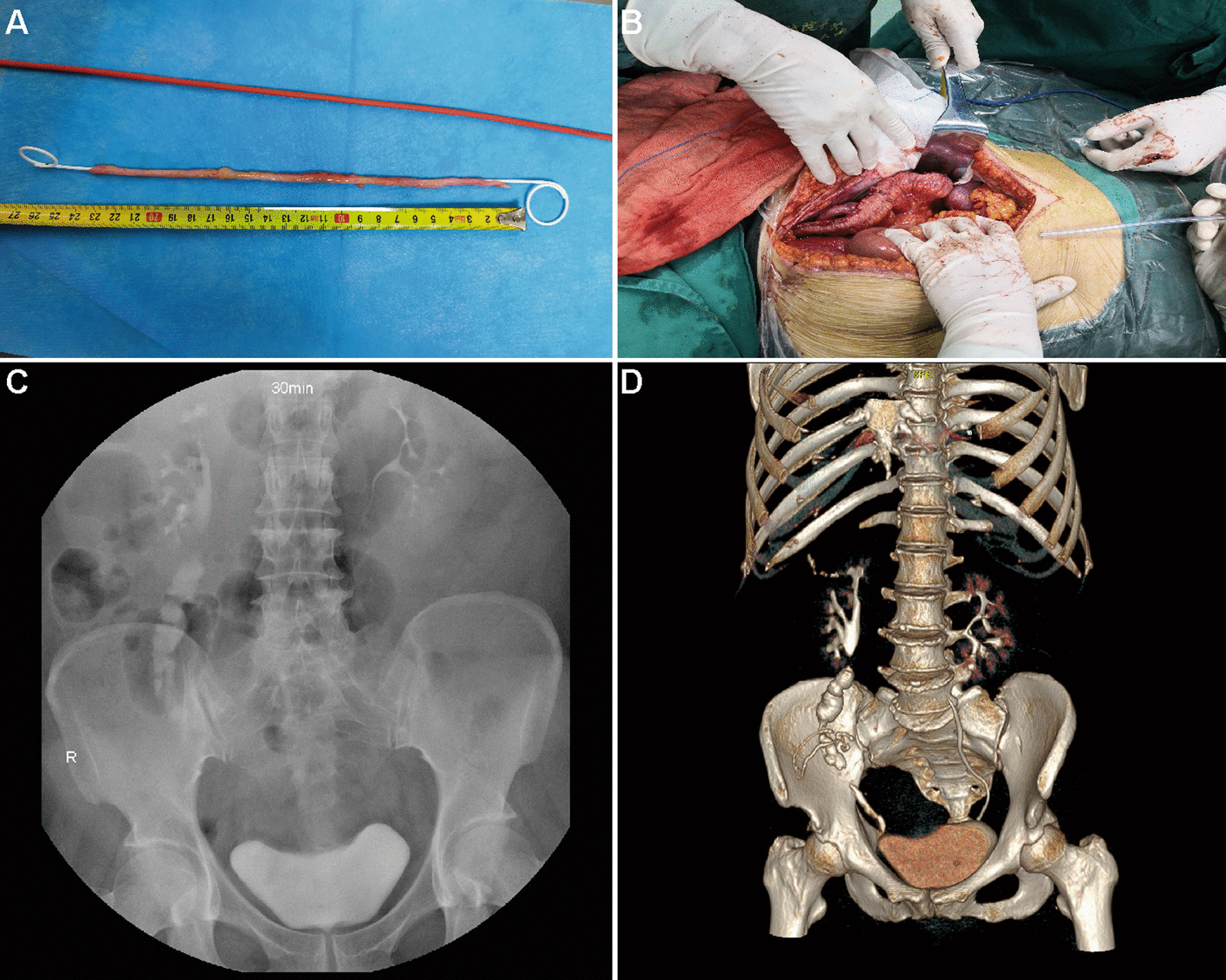
Images of patient 2. **A** Avulsed panureter approximately 23 cm in length. **B** Bridging the ureter by retubularized TDI. **C** Intravenous urogram in months 9 post-surgery. **D** CT urogram in month 27 post-surgery

**Fig. 4:**
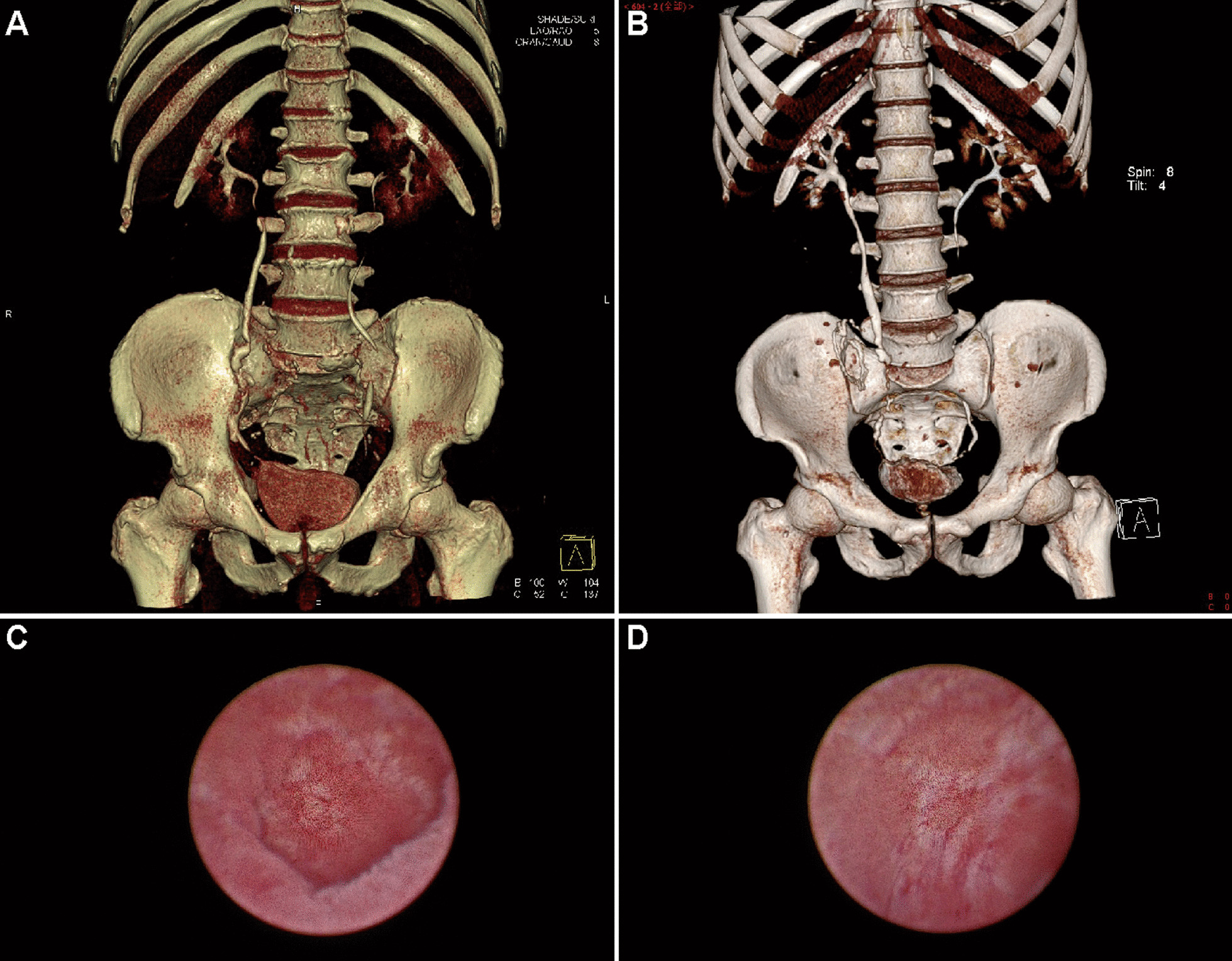
Images of patient 3 and 4. **A** CT urogram of patient 3 in month 36 post-surgery. **B** CT urogram of patient 4 in month 23 post-surgery. **C** Ileal mucosa regrowth of patient 3 in month 30 post-operation. **D** Ileal mucosa regrowth of patient 4 in month 18 post-operation

Mean operation time was 443 min (range 360–550) and blood loss was negligible. All patients’ postoperative urine was reddish in the early 3–5 days but without clotting and related ureteral or vesical obstruction. The mean follow-up was 29 months (range 23–36). According to the Clavien-Dindo classification, postoperative complication grade IIIb occurred in patient 1 who underwent uterine evacuation, repair, and ureter substitution [9]. The patient suffered recurrent high fevers 23 days after surgery. A percutaneous nephrostomy was performed to subside her pyrexia. Reflux pyelonephritis was diagnosed after retrograde urography. Reintervention was required to correct reflux by tapering the distal part of the ileal ureter. Thereafter, the patient recovered smoothly and complied with a regular follow-up (Fig. 2). The other 3 patients recovered uneventfully. The drainage tube was removed on day 5 post-operation and the double J stent removal was on month 3 post-operation. During follow-up, the serum creatinine of the 4 patients didn’t change noticeably. Lumina of neo-ureters remained patent, without stricture formation due to mucosal stripping or ileal wall tapering. No mucus obstruction, calcification, stone formation, or urinary tract tumor recurrence was observed in the period of up to 36-month follow-up (Figs. 3, 4A, B). All the renal units maintained adequate function. Mild hydronephrosis and split renal function impairment occurred in patient 1 as a result of vesicoureteral reflux but remained stable after the repair operation in a 2-year follow-up (Table 1). Intestinal mucosa regrowth was observed 18 months after the procedure. Dissimilar to the long dense finger-like projections of the origin, renewed mucosa presented in the form of short isolated islands (Fig. 4C,D).

## Discussion

The main concern with ileal ureter replacement is the possibility of postoperative complications such as mucous obstruction, metabolic disorder, and renal function deterioration caused by mucous secretion, metabolite absorption of ileal mucosa, and a large cross-sectional diameter of the ileal lumen. To circumvent these risks, we modified ileal segments by tapering the wall and stripping the mucosa and have already conducted an animal study to confirm its safety and efficacy [8]. The purpose of the present study is to further report its feasibility and outcomes in patients. All the patients tolerated the procedure. Medium-term follow-up results indicated that all 4 neo-ureters remained patent, devoid of fibrosis, stricture formation, metabolic disorders, and electrolyte imbalance (1 patient recovered after a repair operation). To the best of our knowledge, this is the first report to document ureteral substitution's safety and medium-term outcomes with TDI in patients with LSUD.

Because of its inherent function of secretion and absorption, ileal mucosa becomes troublesome when used to replace a part of the urinary tract. To overcome the complications it caused, intestine seromuscular grafts were proposed and used in canine enterocystoplasty, but severe shrinkage occurred [10–12]. Several years later, Lima used demucosalized colon or ileum combined with an inflatable silicone model to prevent graft contraction in bladder augmentation and improved the outcomes [13]. Herein, we first adopted demucosalized ileum for lengthy ureteral replacement. In terms of stripping depth, we preferred to use a surgical scissor to only strip the mucosa. According to Dewan’s research, removal of the muscularis mucosa with the inner portion of the submucosa is required to prevent enteric mucosal regeneration [14]; however, this also risks impairing microcirculation and the enteric nervous system of the ileal flap, which accounts for the substitute's shrinkage and contraction [15, 16]. In the ureteroscopic follow-up of our 2 cancer patients, ileal mucosal regrowth did occur in month 18 post-operation. The renewed mucosa, however, became acclimated to the urine environment and was characterized by diminished mucus secretion capacity.

Another vexing issue with ileal replacement is the large cross-sectional diameter. It will cause back-and-forth, retrograde peristalsis, progressive dilatation, and elongation, thereby resulting in urinary stasis, obstruction, and renal function deterioration [17]. The Yang-Monti technique reduces ileal diameter and associated complications. However, this approach necessitates more bowel segments for LSUD, particularly in the panureter defects, which means more anastomoses and a higher risk of postoperative urine leakage and infections. Shokeir first adopted tailored ileal segments for ureteral replacement and achieved better treatment results [18]. Their method mitigated but did not eliminate mucus secretion. We integrated the mucosa stripping technique and ileal tapering technique to restore the continuity of long ureteral defects. Only 1/3 of the ileal wall was tapered in patient 1, and this was still too large for ileal ureter replacement. Afterward, we increased the tapered portion to 1/2–2/3 of its original size, making it more suitable for replacement.

Some researchers set serum creatinine value at 2 mg/dL as a threshold to perform ileal ureter replacement [19]. One of our patients with renal dysfunction, serum creatinine 2.65 mg/ml, did not experience metabolic disorders post-operation, which might indicate the amelioration of absorptive ability. However, the preoperative renal function of the other 3 patients was normal, with the ability to compensate for metabolic disorders. Whether mucosa stripping could be beneficial to reduce absorption remains to be elucidated.

## Conclusions

Ileal wall tapering and mucosa stripping confined to the muscularis mucosae level will not result in shrinkage, fibrosis, or stricture formation of the ileal ureter. Adequate ileal wall tapering, appropriate mucosal stripping, and nipple valve technique are required to reduce postoperative complications. The present work provides evidence for further application of TDI in the replacement of LSUD in patients.

## Data Availability

All data generated or analysed during this study are included in this published article.

## Abbreviations

LSUD: Long-segment ureteral defect
TDI: Tapered demucosalized ileum
UTUC: Upper tract urothelial carcinoma
CT: Computed tomography
